# Socioeconomic and Lifestyle Factors Related to Cost and Frequency of Hospitalization in European Older Adults

**DOI:** 10.3390/ijerph18062833

**Published:** 2021-03-10

**Authors:** Isabel Pardo-Garcia, Elisa Amo-Saus, Pablo Moya-Martinez

**Affiliations:** 1 School of Economics and Business Administration, Castilla-La Mancha University (UCLM), 02071 Albacete, Spain; isabel.pardo@uclm.es; 2 Sociosanitary Research Center, 16071 Cuenca, Spain; pablo.moya@uclm.es; 3 Research Group of Economy, Food and Society, Castilla La Mancha University, 02071 Albacete, Spain; 4 School of Social Sciences, Castilla- La Mancha University (UCLM), 16071 Cuenca, Spain

**Keywords:** health care utilization, costs, economics of ageing, physical activity

## Abstract

Individuals’ lifestyles play an important role in healthcare costs. A large part of these costs is derived from hospitalizations. With the aim of determine the relationship between lifestyle and the likelihood of hospitalization and associate costs in older adults, this study used the Survey of Health, Aging, and Retirement in Europe. Generalized regression models for panel data were developed and adjusted hospitalization costs derived from the length of hospital stay were also estimated. The average adjusted cost of hospitalization was I$ 9901.50 and the analyses showed that performing weekly physical activity significantly reduces the probability of hospitalization (OR: 0.624) and its costs (I$ 2594.5 less per person per year than subjects who never performed physical activity). Muscle strength plays an important role in this relationship and eating habits are not of great significance. Furthermore, we found interesting differences in the frequency and costs of hospitalization between subjects by country.

## 1. Introduction

Population aging influences the design and implementation of healthcare policies. The demographic structure of Europe changed over the 20th century and will continue to do so in the coming decades. By 2070, 30% of people in Europe are estimated to be aged 65 and above, up from about 20% today. From 2019 to 2070, the share of people aged 80 or over is projected to more than double, reaching 13% [1].

This increase in the older population has a great impact on health systems [2] and their sustainability [3], as reflected by the progressive rise in demand for services [4,5]. As people age, the likelihood of developing disabilities and comorbidities increases. For example, various authors showed that older adults consume more health resources than the general population [6,7,8]. According to Hewitt et al., older persons suffer multimorbidity, explaining 74% of health resource consumption [9]. Other authors have also shown that comorbidities increase total health costs and the cost of such patients is higher [10,11,12,13].

Additionally, there is a growing prevalence of chronic illnesses, such as diabetes or cardiovascular disease; such diseases cause disabilities and reduce life expectancy, with their impact on healthcare expenditure expected to increase in coming years [14]. These diseases are associated with the environment and genetic factors, but also with inappropriate lifestyle factors, such as unhealthy diet and low physical activity [15,16,17], and thus lifestyles impact the use of healthcare services. These two factors are responsible for two of every five deaths in the world and account for 30% of overall world illness burden [18,19].

The literature includes studies on the burden, the cost of diseases and factors related to healthcare resource consumption [5,6,18,19,20]. However, in the field of the analysis of outpatient visits, emergency care, and hospitalization, there are specific studies on costs associated with (1) psychiatric diseases [21,22,23]; (2) the existence of social support networks [24]; (3) heart failure [25,26,27]); (4) diabetes [28] and osteoarthritis [29]; (5) bone fracture in older adults [30,31]; or (6) the influence of physical activity on length of hospital stay after surgery [32], but the impact of lifestyle factors, such as dietary habits or level of physical activity, in older persons has been the subject of less study, despite physical inactivity being the fourth risk factor for mortality in the older population [33].

The work by Sari, for example, demonstrated the relationship between physical activity and the use of healthcare services in older adults, although the variations in their methodology advised further studies be conducted [16]. Cantarero-Prieto et al. analyzed the effect of lifestyle in countries in the south of Europe [34]. In this sense, it is important to extend the analysis to other European countries and to determine the factors that affect the probability of hospitalization.

So, the aim of the present work was to determine the relationship between lifestyle and the likelihood of hospitalization in European older adults. We also aimed to analyze the costs and length of hospitalizations, and whether there exist differences in sociodemographic variables as regards the probability of hospitalization and the associated costs across the European countries analyzed.

## 2. Material and Methodology

We drew upon the Survey of Health, Aging, and Retirement in Europe (SHARE). The survey addresses persons aged 50 years and over who reside in the respective country of interview. Furthermore, SHARE consists of six waves (the third of these being different because it focuses on people’s life histories) from 2004 to 2015. As each wave includes new variables, countries and other improvements, we used the 2011 and 2013 waves, which had homogeneous data on the variables necessary for our analysis. More information on the data used, and the survey can be found in Release Guide 6.0.0 [35]. From these waves, and as inclusion criteria, we selected the individuals that responded in both 2011 and 2013. A total of 58,184 subjects were studied in the first wave and 66,221 in the second. Finally, and with the aim of assessing the increase/decrease in the number of hospitalizations after two years of follow-up, we selected the individuals that had completed the 2 surveys (38,376).

### 2.1. Dependent Variables

The main dependent variables in our analysis were the frequency of hospitalization and the cost of hospitalization per person and year. For the first variable, the survey includes a question about how many times the respondent has been admitted to a hospital for at least one night in the last 12 months and another for the number of days per stay. To obtain the cost of hospitalization, we multiplied the total number of days in hospital by the cost per day of hospitalization. This daily cost was obtained for each country from the econometric estimates of unit costs made by the World Health Organization (WHO), which considered the cost per day and bed in a public primary hospital [36]. These costs do not include drugs and diagnostic tests but do cover costs such as staff, capital, and food. Finally, for each country, the costs were converted to 2008 international dollars, reflecting the current year’s exchange rates and current Purchasing Power Parity (PPP) adjustments. These may also be adjusted for inflation to represent currencies in constant (international) dollars for a base year, such as 2008. Harmonized Indices of Consumer Prices (HICPs) published by Eurostat for each country were used to update unit costs to 2013 [37].

### 2.2. Independent Variables

The sociodemographics of sex, age, and marital status were included as independent variables The equivalent income per household was obtained, adjusting for household size by dividing the income by 1 for the first adult in the household plus 0.5 for each adult, following the OECD-modified scale proposed by Hagenaars et al. [38]. Given the characteristics of the sample, the weighting per resident child was not used. This variable is sufficient according to previous studies, which have evidenced the good performance of this indicator in capturing health variation in older ages and its robust link to measuring individuals’ health [39]. Finally, we performed a logistic transformation of the income. It is worth noting also that the number of years that the subject had been studying was analyzed and no significant differences were found, and so this variable was not included in the models.

As regards the variables related to an individual’s health, we used the number of comorbidities, based on the comorbidity index included in the survey. This variable was recoded into three levels: no comorbidities, medium comorbidity (1–2), and high comorbidity (3–8). Body Mass Index (BMI) is defined as a person’s weight in kilograms divided by the square of their height in meters (kg/m^2^). This was based on self-reported weight and height. Muscular strength was obtained from the survey data on the maximum measure of grip strength. For this variable, two measurements on each hand were recorded using a dynamometer. Valid measurements are defined when the two measurements of one hand do not differ by more than 20 kg. If the difference was above that limit and/or was only measured once on one hand, the measurements were recoded as missing. However, two valid measurements on one hand were included. Grip strength measurements of zero or above 100 kg were also recoded as missing. This variable was subsequently recoded into tertiles of muscular strength, with the higher the tertile, the greater the muscular strength.

The variables of individuals’ habits as regards moderate and vigorous physical activity were recoded into never, once a week or less and more than once a week. Smoking and alcohol consumption were coded as yes and no dummy variables.

We also obtained data on variables related to dietary habits using the survey questions referring to how often the participant consumed different types of food in a normal week. The categorical variables were recoded as <1 times a week, 1–2 times a week, and 3–6 times a week, for fruit or vegetables, dairy products (i.e., a glass of milk, cheese in a sandwich, a pot of yogurt, a can of high protein supplement), legumes, beans, egg consumption, and meat, fish, or poultry consumption.

Finally, a dummy variable was included on country of origin with the aim of comparing differences in hospitalizations and costs between countries. Different analyses were performed with different recodifications on many variables. We decided to use the more parsimonious recodification to have more simple models. In addition, possibly due to education systems differing across the countries in the survey, a question was included on how many years the respondent had been in full time education. We tested this variable in the model. As it was found to be non-significant, we considered it more appropriate to use the variable of equivalent income per household to capture the effects of an individual’s socioeconomic status and education level.

### 2.3. Statistics

First, a descriptive analysis was performed to determine the main characteristics of the sample. Then Generalized Linear Models for Panel Data (GLM-PD) with logic link function were used with hospitalization frequency as the dependent variable. These models are widely used in data with bias in the distribution, such as frequency of hospitalization or hospitalization costs [40]. Different models were tested to check the robustness of the results. Important variables were identified, as were significant differences between countries, so we decided to implement the same model, but change the country of reference, in order to identify the size of the difference in the likelihood of hospitalization across countries adjusted for the other variables. This information might give a picture of the differences between countries in variables not measured in the study, such as access to hospital resources for the population over the age of 50 or the lack of services at this level of health care.

Finally, the impact of lifestyle factors on annual hospitalization costs was estimated using GLM-PD with gamma link function. It is worth highlighting here that hospitalization costs include the number of times an individual is hospitalized, the length of the hospital stay in days, and, in order to harmonize findings across countries, the unit costs per day (also previously discussed) adjusted using Purchasing Power Parity (PPP), which allows for comparison between countries after taking into account differences in purchasing power.

For the analyses, we used R Studio with R version 4.0.2. and Stata IC/16.1.

## 3. Results

We analyzed data on 38,376 individuals resident in countries of the European Union. Estonia was the country that contributed the largest number of respondents to the sample (14%), while residents in Germany accounted for the lowest proportion. Table 1 presents a descriptive analysis by country and for the different waves, as well as the increase or decrease in hospitalizations over the last 12 months. In the variables of sex and age, no considerable differences were found between countries, with 57% of women and a mean age of 66.12 years (SD: 9.726) for the overall sample.

A total of 85% of the respondents in 2011 had not been hospitalized over the last twelve months. In all the countries, except Italy, there was a drop in the number of individuals that had not been hospitalized, finding that an approximately two-year increase in the mean age of the sample is related to a 1.5% rise in hospitalization over the previous 12 months. Spain, Estonia, and Slovenia presented the highest increase in the rate of hospitalizations (2.5–3.1%), while France, Belgium, and the Netherlands showed the lowest increases (0.9–1%). The mean frequency of hospital stay was 0.248 (SD: 0.845) per person for 2011, with a mean increase of 0.033 in 2013, while the mean frequency of hospital stay for individuals that had been hospitalized at least once was 1.684 (SD: 1.559) and 1.730 (SD: 1.578) for 2011 and 2013, respectively. No sizeable differences were found between countries in the means under analysis, except in the case of Slovenia, where the mean hospitalization rate in 2011 was 2.746 (SD: 3.023).

Table 2 shows the odds ratios from the generalized linear models for panel data, showing the factors that have the greatest effect on hospitalization and their effect size. Model 1, in which we did not adjust by country, shows that being female, having good muscular strength (second or third tertile) and engaging in physical activity, either moderate or vigorous (compared to not doing any) are important protective factors against hospitalization. Consuming legumes, beans, or eggs more than once a week was also found to be a protective factor, but with a smaller effect size (significance of 95%). Additionally, being married and presenting medium or high comorbidity notably increase this risk. Age, BMI, smoking, drinking alcohol, and other dietary habits (consumption of dairy products, fruit, or vegetables or meat, fish, or poultry) failed to show significance.

Model 2 included all the countries, and we discarded the effect of muscular strength, finding similar coefficients in all the factors, although the consumption of legumes, beans, and eggs and the equivalized income per person did not reach the level of significance. This model also shows a slight increase to 95% in significance of smoking. In addition, we found a significant effect of age in individuals of over 70 years, with a relationship found between this factor and muscular strength. In Model 3, where the factors associated with physical activity were also excluded, it can be seen that BMI takes on the effects of these factors, showing a slight effect (significance of 95%). We also included as protective factors (although not of great magnitude) equivalized income, not drinking alcohol and the consumption of legumes, beans and eggs. The fourth and last model included all the variables and showed the importance of those related to physical activity and muscular strength, compared to dietary habits, unhealthy habits, and age. The odds ratios for engaging in vigorous or moderate physical activity are highly similar, with it being more beneficial to do physical activity more than once a week.

Table 3 shows the odds ratios of hospitalization by country from the GLM-PD, which can be constructed by changing the reference country in the country variable. The objective of this analysis is to quantify the differences in the probability of hospitalization between each of the countries after adjusting for the study variables.

This table helps explain that living in Spain is a protective factor against hospitalization compared to living in Austria with an odds ratio of 0.285. On the other hand, living in the Czech Republic increases the probability of hospitalization 2.155 times more than in Spain.

Furthermore, is necessary to indicate that since these probabilities are adjusted for the rest of the variables of the model, the differences are arguably due to omitted variables, such as, for example, different access to healthcare, but we cannot determine the precise reason for the differences.

**Figure 1:**
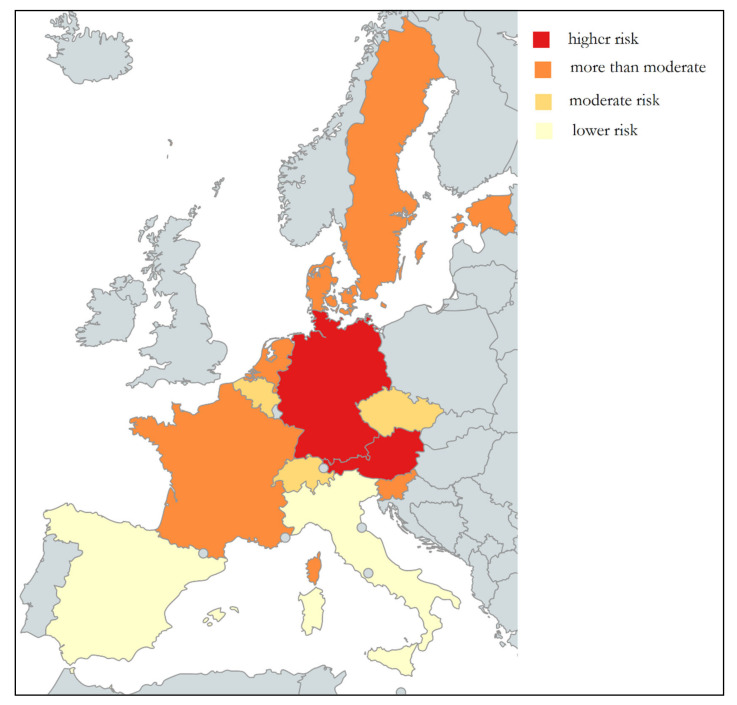
Adjusted risk of hospitalization in analyses countries. Gray color indicated no country analyzed. Darker colors indicate higher risk.

Broadly speaking and taking into account a significance level of 99%, we could classify the thirteen countries in four groups according to risk of hospitalization. The first group, that with the highest risk, comprises Germany and Austria, followed by a large group formed by Sweden, Netherlands, France, Denmark, Estonia, and Slovenia. The third group according to likelihood of hospitalization is composed of Czech Republic, Switzerland, and Belgium, while the group with the lowest risk is formed by Spain and Italy. This suggests, once the figures are adjusted for the differences in purchasing power and other individual characteristics individuals (health, diet, and lifestyle habits), that variations in hospitalization rates are the result of differences in the health systems. Figure 1 shows a map with the different risk groups.

Table 4 shows the GLM-PD that explain the variables affecting hospitalization costs. In the same line as the models in Table 2, these include different variables, but show a similar pattern of significance, although intis case there are no differences by gender. The factors associated with physical activity and muscular strength show greater importance than dietary habits, with it being worth noting that hospitalization costs for individuals doing moderate physical activity at least once a week decrease by I$ 2494.50 (per person), compared to those that perform no physical activity. As for the adjusted hospitalization costs between countries, the highest are between Austria, Germany, and Switzerland, compared to the rest.

## 4. Discussion

This study examines the costs and frequency of hospitalization in older adults in Europe and their association with lifestyle factors. The mean cost of hospitalization per person was I$ 5145 per year. When adjusted for the other variables, this cost increases to around I$ 9000, while engaging in physical activity can reduce this cost by I$ 2500.

Our initial findings showed that as age increases, so rises the likelihood of hospitalization, which is consistent with previous studies suggesting that older persons consume more healthcare resources [6,7,8]. Furthermore, our study showed that, without taking into account the residence country, having a medium or high comorbidity increases the risk of hospitalization, which is consistent with the findings of Suarez-García et al. [4] and Ku et al. [2]. In a different setting, a study conducted in the USA concludes that multimorbidity also increases the likelihood of hospitalization leading to higher costs [13], which is similar to the findings of a study carried out in Switzerland for elderly population [11].

However, in the models adjusted for muscular strength and physical activity, age is no longer significant, underlining the key nature of promoting engagement in physical activity and the development of strong muscles.

We also found differences by country in likelihood of hospitalization, coinciding with the work by Srakar et al. [41]. The variation in hospitalization rates could be the result of differences in the health system. The countries selected present differences in the public or private provision of the health services, for example, in the filters to use emergency services or to visit a specialist, while for some services a copayment is required, among others [42].

The analysis of the impact of lifestyle habits on the frequency and cost of hospitalization revealed no significant findings for smoking, drinking alcohol or BMI. Smoking is currently seen as one of the most important single risks to health, and is responsible for a large financial burden on healthcare systems [43]. Our findings reveal that if we rule out the effect of muscle strength, smoking shows a significant effect. In the same line, a study in a population of individuals older than 18 years [44] shows that cigarette smoking is associated with higher health care utilization (frequent hospitalization and outpatient visits) for current and former smokers compared to people who have never smoked, which translates into higher medical costs. Another study in people over 35 [45] shows that reducing smoking can lower the associated health care burden. Moreover, smoking can increase overnight hospital stays by 14.22% [34].

In contrast, when we adjust for all variables, the study shows that smoking is not predictive of the use of health services, perhaps because our study was carried out in a population older than 50 years and health problems increase with aging, while not drinking and not smoking are adaptations after health problems [5].

As for drinking, even though The World Health Organization states that alcohol consumption causes a great health, social, and economic burden [33], it is also true that previous studies show that, on average, light to moderate drinkers of alcohol appear to be in better health, both mentally and physically and have better functional status, compared to abstainers or heavy drinkers [46,47,48]. Moreover, since our study only considers the options of “alcohol drinker” or “non-alcohol drinker”, our results coincide with these studies; that is, drinking alcohol is not necessarily associated with a worse state of health [49]. As regards BMI, in other life stages, it can be a significant predictor of obesity, and is thus associated with a greater number of diseases and the likelihood of increasing health expenditure. However, older adults typically present a loss of lean mass, which might mask obesity through a drop in BMI. Thus, we posit other measures, such as those for muscular strength, as better indicators [50].

Furthermore, as mentioned, our study shows that both individuals with good muscle tone and those who engage in vigorous or moderate physical activity more than once a week, can reduce the mean cost of hospitalization, with these variables acting as protective factors against the risk of hospitalization. These findings are in line with those of previous studies. Daher et al. found that older adults who did physical activity had a better quality of life and made less use of hospital and emergency services [51]. Cantarero et al. found that visits to the doctor and hospital admissions were lower in those engaging in physical activity [34]. Sari reported that lack of physical activity increased the length of hospital stays and the use of medical services [52]. Additionally, Haveman-Nies et al. found that, broadly speaking, individuals that did physical exercise enjoyed better health [53].

These findings also coincide with the WHO’s recommendations on physical activity and with the evidence that the health of individuals that engage in physical activity is better [54]. Moreover, considering physical activity as intimately linked to diet, nutrition and health is key, because it specifically affects fat, muscle, and bones, which ultimately impacts frequency of hospitalization and recovery [17,54]. The importance of physical activity in maintaining muscle mass was reported by Paterson et al. [55]. Indeed, its benefits include improvements in cardiorespiratory condition, strength and, indirectly, weight, all components negatively affected in older persons due to their physical limitations in daily life activities. In this line, maintaining muscular mass is important to lower the risk of fractures and reduce fragility [56,57]. Nutrition also has an impact on muscular tone. The presence of malnutrition or nutritional risk also enhances the risk of hospitalization [58] and increases healthcare expenditure [59,60]. In our study, this may not be especially significant as, broadly speaking, in the countries analyzed, there is not a sufficient deficit in any of the categories described or whose impact is significant enough to increase hospitalization rates. The study by López Gimenez et al. concluded that diet does not appear to be associated with either better or worse health state [61]. It is also possible that reduced calorie intake (a variable we were unable to study) might be more important than dietary habits from a certain age. Moreover, the interactions between different dietary factors were analyzed but revealed no significant relationships.

Understanding the factors influencing health-service use in older adults and following our results, the design of public policies of prevention is the strength of our study.

It is also important to indicate some limitations of the present study. Although we consider the sample is large enough to be representative, some subjects, for various reasons beyond our knowledge (change of country of residence, death, refusal to continue participating, not being located, etc.) likely did not complete the second wave and, therefore, this might generate a bias, but given the diversity of cases we consider it should not be of great magnitude. It is worth mentioning that the sample distribution by country shows a low percentage of subjects in Germany (2.85%) and Sweden (3.88%). Excluding these countries, the mean percentage per country was 8.48 ± 2.86.

Furthermore, it is well known that Europe is a region of immigrants. In the relationship between lifestyle and probability of hospitalization, immigrant status is a variable of interest. In the survey, we found a question about the place of birth of the subjects that is independent of the country of the survey, but it is not possible to distinguish between those who are recent immigrants and those immigrated almost a lifetime ago. Therefore, we consider this variable could be a confounding factor.

Other variables of interest such as occupation, region of residence (urbanization) and religion, must be analyzed in future studies. Results in health are related to the type of health care model and individuals’ habits. Because we have only considered individual habits and since the country of residence was also found to be a significant factor, additional studies considering health systems should be developed. These future studies would make it possible to unravel part of these differences between countries evidenced in our study and to attempt to adjust these inequities by implementing policies.

## 5. Conclusions

We have sought to determine how lifestyles affect the likelihood of hospitalization in European older adults, in addition to analyzing the costs and length of hospitalizations. Specific, empirical evidence is included on the differences between socioeconomic factors and the probability of hospitalization and associated costs in several European countries. Our results highlight that, in population aged 50 or older, age, BMI, unhealthy habits, and dietary habits in general failed to show significance, while physical activity is confirmed as the determining habit for reducing healthcare costs. These findings should be incorporated into health campaigns promoted by countries’ governments.

## Figures and Tables

**Table 1:** Descriptive statistics of the study sample and times hospitalized by country.

					Times Hospitalized								
					2011			2013			Δ(2011–2013)		
Country	*n*	%	Age Mean (SD)	Female %	Zero Times %	Mean (SD)	Mean Censured (SD) *n* = 5630	Zero Times %	Mean (SD)	Mean Censured (SD) *n* = 6220	Zero Times %	Mean (SD)	Mean Censured (SD) *n* = 10,097
Austria	3992	10.40	65.73 (9.638)	57.29	78.35	0.336 (0.854)	1.551 (1.220)	76.88	0.389 (0.962)	1.683 (1.350)	−1.47	0.055 (1.161)	0.151 (1.917)
Germany	1094	2.85	67.34 (8.233)	52.83	80.22	0.348 (0.983)	1.759 (1.551)	78.59	0.335 (0.851)	1.564 (1.211)	−1.63	−0.012 (1.264)	−0.034 (2.15)
Sweden	1488	3.88	69.44 (8.497)	54.64	86.05	0.205 (0.704)	1.469 (1.306)	84.73	0.247 (0.762)	1.621 (1.258)	−1.32	0.040 (0.938)	0.157 (1.847)
Netherlands	2233	5.82	65.81 (9.224)	56.29	88.95	0.169 (0.682)	1.533 (1.459)	87.98	0.193 (0.706)	1.608 (1.368)	−0.98	0.024 (0.897)	0.117 (1.985)
Spain	2959	7.71	67.87 (10.526)	55.53	88.89	0.204 (0.822)	1.838 (1.758)	85.76	0.232 (0.755)	1.631 (1.313)	−3.13	0.029 (1.007)	0.133 (2.162)
Italy	2578	6.72	66.77 (9.043)	55.16	87.37	0.182 (0.576)	1.440 (0.903)	88.83	0.198 (0.773)	1.770 (1.605)	1.46	0.016 (0.884)	0.074 (1.931)
France	3912	10.19	66.01 (10.367)	57.00	85.12	0.281 (0.978)	1.888 (1.843)	84.19	0.324 (1.124)	2.047 (2.115)	−0.92	0.045 (1.384)	0.169 (2.686)
Denmark	1922	5.01	64.55 (10.042)	53.80	88.78	0.180 (0.657)	1.605 (1.252)	86.81	0.213 (0.765)	1.617 (1.474)	−1.97	0.033 (0.886)	0.156 (1.911)
Switzerland	2890	7.53	65.14 (9.694)	54.53	86.95	0.236 (0.893)	1.806 (1.809)	85.68	0.255 (0.910)	1.785 (1.749)	−1.26	0.019 (1.193)	0.081 (2.431)
Belgium	3900	10.16	65.33 (10.263)	55.67	83.92	0.238 (0.722)	1.482 (1.184)	82.87	0.271 (0.795)	1.580 (1.276)	−1.05	0.031 (0.974)	0.112 (1.846)
Czech Republic	4005	10.44	65.6 (8.833)	59.05	82.79	0.304 (0.973)	1.769 (1.706)	81.24	0.360 (1.110)	1.919 (1.891)	−1.54	0.055 (1.384)	0.18 (2.502)
Slovenia	1942	5.06	65.82 (9.928)	57.05	87.60	0.340 (1.396)	2.746 (3.023)	85.14	0.248 (0.846)	1.670 (1.566)	−2.46	−0.093 (1.599)	−0.388 (3.259)
Estonia	5461	14.23	66.33 (9.657)	60.76	86.67	0.202 (0.635)	1.515 (1.018)	83.79	0.272 (0.867)	1.680 (1.508)	−2.87	0.070 (0.981)	0.276 (1.930)
Total	38,376		66.12 (9.726)	56.81	85.28	0.248 (0.845)	1.684 (1.559)	83.74	0.281 (0.901)	1.730 (1.578)	−1.54	0.033 (1.141)	0.126 (2.216)

SD: Standard Deviation; For the difference of mean censured, subjects with zero times in the initial or final wave are excluded.

**Table 2:** Panel Estimators of hospitalization for Generalized Linear Models with logit family.

	Model 1	Model 2	Model 3	Model 4
(Intercept)	0.108 (0.078; 0.148) ***	0.287 (0.209; 0.393) ***	0.137 (0.1; 0.188) ***	0.313 (0.223; 0.440) ***
Female	0.816 (0.772; 0.863) ***	0.808 (0.766; 0.852) ***	0.853 (0.809; 0.9) ***	0.813 (0.770; 0.859) ***
Age (ref. 50–60 y)				
60–70 y	0.963 (0.898; 1.033)	0.996 (0.932; 1.065)	1.033 (0.966; 1.105)	0.945 (0.881; 1.014)
70–80 y	1.075 (0.994; 1.162)	1.204 (1.12; 1.295) ***	1.363 (1.269; 1.465) ***	1.037 (0.959; 1.121)
80 year and more	1.091 (0.985; 1.208)	1.3 (1.187; 1.423) ***	1.777 (1.627; 1.942) ***	1.039 (0.938; 1.150)
Marital status (ref. other situations)				
Married	1.132 (1.068; 1.199) ***	1.082 (1.024; 1.144) **	1.103 (1.044; 1.166) **	1.064 (1.004; 1.127) *
Comorbidity (ref. no comorbidity)				
Medium	1.949 (1.829; 2.075) ***	1.981 (1.865; 2.105) ***	2.069 (1.948; 2.198) ***	1.942 (1.824; 2.069) ***
High	3.659 (3.370; 3.972) ***	3.732 (3.453; 4.034) ***	4.301 (3.98; 4.647) ***	3.598 (3.314; 3.906) ***
BMI	1.005 (0.999; 1.01)	0.999 (0.994; 1.004)	1.005 (1; 1.011) *	1.004 (0.999; 1.010)
Muscle strength (ref. 1st tertile)				
Second tertile	0.772 (0.725; 0.822) ***			0.730 (0.685; 0.778) ***
Third tertile	0.672 (0.624; 0.722) ***			0.613 (0.570; 0.660) ***
Vigorous physical activities (ref. Never)				
Once a week or less	0.752 (0.684; 0.826) ***	0.685 (0.626; 0.750) ***		0.722 (0.657; 0.794) ***
More than ones a week	0.703 (0.662; 0.745) ***	0.627 (0.592; 0.664) ***		0.675 (0.636; 0.716) ***
Moderate physical activities (ref. Never)				
Once a week or less	0.733 (0.649; 0.828) ***	0.667 (0.596; 0.745) ***		0.709 (0.628; 0.801) ***
More than ones a week	0.643 (0.593; 0.697) ***	0.578 (0.538; 0.621) ***		0.624 (0.576; 0.676) ***
Log income	1.045 (1.024; 1.065) ***	0.994 (0.973; 1.015)	0.977 (0.957; 0.997) *	0.995 (0.973; 1.018)
No smoking	1.035 (0.964; 1.110)	1.078 (1.008; 1.154) *	1.018 (0.951; 1.089)	1.062 (0.990; 1.139)
No drinking	0.962 (0.896; 1.033)	0.944 (0.881; 1.011)	0.906 (0.846; 0.972) **	0.938 (0.873; 1.007)
Dairy products (ref. <1 times week)				
1–2 times a week	0.985 (0.860; 1.128)	0.965 (0.849; 1.096)	0.945 (0.832; 1.074)	0.973 (0.850; 1.113)
3–6 times week/every day	0.974 (0.878; 1.079)	0.950 (0.861; 1.048)	0.913 (0.827; 1.007)	0.961 (0.866; 1.065)
Legumes, beans or eggs (ref. <1 times week)				
1–2 times a week	0.919 (0.863; 0.978) **	0.952 (0.897; 1.011)	0.935 (0.881; 0.993) *	0.968 (0.909; 1.031)
3–6 times week/every day	0.918 (0.864; 0.975) **	0.978 (0.922; 1.038)	0.956 (0.900; 1.014)	0.995 (0.935; 1.06)
Fruits or vegetables (ref. <1 times week)				
1–2 times a week	1.076 (0.889; 1.303)	1.057 (0.883; 1.266)	1.023 (0.855; 1.225)	1.072 (0.886; 1.296)
3–6 times week/every day	0.998 (0.855; 1.164)	1.064 (0.920; 1.232)	0.980 (0.847; 1.134)	1.043 (0.894; 1.217)
Meat, fish or poultry (ref. <1 times week)				
1–2 times a week	1.029 (0.911; 1.163)	1.057 (0.943; 1.185)	1.057 (0.943; 1.185)	1.060 (0.938; 1.198)
3–6 times week/every day	0.912 (0.819; 1.015)	0.972 (0.878; 1.075)	0.959 (0.867; 1.062)	0.990 (0.889; 1.103)
Country (ref. Austria)				
Germany		0.851 (0.732; 0.99) *	0.833 (0.715; 0.969) *	0.880 (0.754; 1.028)
Sweden		0.566 (0.488; 0.656) ***	0.545 (0.470; 0.633) ***	0.589 (0.506; 0.686) ***
Netherlands		0.464 (0.406; 0.531) ***	0.459 (0.402; 0.525) ***	0.477 (0.415; 0.548) ***
Spain		0.335 (0.295; 0.381) ***	0.394 (0.347; 0.447) ***	0.285 (0.248; 0.327) ***
Italy		0.334 (0.294; 0.379) ***	0.383 (0.337; 0.435) ***	0.325 (0.283; 0.372) ***
France		0.540 (0.486; 0.600) ***	0.597 (0.537; 0.663) ***	0.517 (0.463; 0.578) ***
Denmark		0.483 (0.421; 0.556) ***	0.486 (0.423; 0.559) ***	0.500 (0.433; 0.577) ***
Switzerland		0.599 (0.533; 0.673) ***	0.603 (0.536; 0.678) ***	0.601 (0.532; 0.678) ***
Belgium		0.600 (0.541; 0.666) ***	0.657 (0.592; 0.729) ***	0.601 (0.539; 0.670) ***
Czech Republic		0.615 (0.554; 0.683) ***	0.627 (0.565; 0.697) ***	0.614 (0.549; 0.686) ***
Slovenia		0.474 (0.415; 0.542) ***	0.474 (0.414; 0.542) ***	0.468 (0.406; 0.539) ***
Estonia		0.475 (0.429; 0.525) ***	0.472 (0.427; 0.521) ***	0.476 (0.428; 0.529) ***

Ref.: reference category; Married refers to ‘Married and living together with spouse’ or ‘Registered partnership’ and the reference category (Other situations) includes: ‘Married, not living with spouse’, ’Divorced’, ‘Widowed’ or ‘Never married’; Odds Ratio and 95% Confidence Interval in brackets; * *p* < 0.05; ** *p* < 0.01;*** *p* < 0.001.

**Table 3:** Countries odds ratio of logit panel model changing reference country for hospitalization in the last 12 months.

Reference Country	Germany	Sweden	Netherlands	Spain	Italy	France	Denmark	Switzerland	Belgium	Czech Republic	Slovenia	Estonia
Austria	0.88 (0.754; 1.028)	0.589 (0.506; 0.686) ***	0.477 (0.415; 0.548) ***	0.285 (0.248; 0.327) ***	0.325 (0.283; 0.372) ***	0.517 (0.463; 0.578) ***	0.5 (0.433; 0.577) ***	0.601 (0.532; 0.678) ***	0.601 (0.539; 0.67) ***	0.614 (0.549; 0.686) ***	0.468 (0.406; 0.539) ***	0.476 (0.428; 0.529) ***
Germany	1	0.669 (0.554; 0.808) ***	0.542 (0.453; 0.648) ***	0.323 (0.27; 0.387) ***	0.369 (0.308; 0.441) ***	0.587 (0.5; 0.689) ***	0.568 (0.473; 0.682) ***	0.682 (0.576; 0.807) ***	0.682 (0.582; 0.801) ***	0.697 (0.593; 0.819) ***	0.531 (0.442; 0.638) ***	0.54 (0.462; 0.632) ***
Sweden		1	0.81 (0.681; 0.963) *	0.484 (0.406; 0.576) ***	0.551 (0.462; 0.658) ***	0.878 (0.753; 1.024)	0.849 (0.712; 1.014)	1.02 (0.866; 1.2)	1.02 (0.875; 1.19)	1.042 (0.889; 1.221)	0.794 (0.663; 0.952) *	0.808 (0.693; 0.942) **
Netherlands			1	0.597 (0.507; 0.703) ***	0.681 (0.577; 0.802) ***	1.084 (0.942; 1.248)	1.049 (0.888; 1.239)	1.259 (1.084; 1.463) **	1.26 (1.095; 1.449) **	1.287 (1.113; 1.487) **	0.981 (0.828; 1.161)	0.998 (0.868; 1.147)
Spain				1	1.14 (0.972; 1.336)	1.816 (1.579; 2.089) ***	1.756 (1.485; 2.078) ***	2.109 (1.808; 2.46) ***	2.11 (1.832; 2.43) ***	2.155 (1.872; 2.481) ***	1.642 (1.393; 1.937) ***	1.671 (1.462; 1.91) ***
Italy					1	1.593 (1.383; 1.836) ***	1.541 (1.3; 1.826) ***	1.85 (1.587; 2.156) ***	1.851 (1.608; 2.131) ***	1.891 (1.647; 2.17) ***	1.441 (1.224; 1.696) ***	1.466 (1.282; 1.676) ***
France						1	0.967 (0.837; 1.118)	1.161 (1.024; 1.317) *	1.162 (1.038; 1.3) **	1.187 (1.055; 1.335) **	0.904 (0.781; 1.048)	0.92 (0.823; 1.029)
Denmark							1	1.201 (1.029; 1.401) *	1.201 (1.04; 1.387) *	1.227 (1.057; 1.425) **	0.935 (0.786; 1.112)	0.951 (0.823; 1.099)
Switzerland								1	1.001 (0.884; 1.132)	1.022 (0.895; 1.167)	0.779 (0.665; 0.912) **	0.792 (0.697; 0.901) ***
Belgium									1	1.021 (0.91; 1.147)	0.778 (0.672; 0.901) **	0.792 (0.708; 0.886) ***
Czech Republic										1	0.762 (0.66; 0.88) ***	0.775 (0.698; 0.861) ***
Slovenia											1	1.017 (0.885; 1.169)
Estonia												1

Odds Ratio and 95% Confidence Interval in brackets; * *p* < 0.05; ** *p* < 0.01; *** *p* < 0.001; Models to obtain odds ratios between countries include female, aged, married, comorbidity, muscle strength, BMI, vigorous physical activity, moderate physical activity, log of income equivalent, smoking, drinking and consumption of dairy products, legumes, bean and eggs, verdures, and vegetables and meat, fish, and poultry.

**Table 4:** Panel Estimators of hospitalization costs for Generalized Linear Models with gamma family.

	Model 1	Model 2	Model 3	Model 4
(Intercept)	6772.6 (5283.2; 8262.1) ***	10,403.2 (8752.0; 12,054.5) ***	7849.5 (5957.5; 9741.4) ***	9901.5 (8197.9; 11,605.2) ***
Female	−197.9 (−447.2; 51.3)	−204.7 (−434.4; 24.9)	−178.4815 (−452.5; 95.5)	−179.9 (−409.4; 49.5)
Age (ref. 50–60 y)				
60–70 y	−7.5 (−305.3; 290.2)	−79.2 (−353.3; 194.9)	103.6 (−223.4; 430.8)	−106.8 (−380.4; 166.7)
70–80 y	52.7 (−295.9; 401.5)	279.5 (−36.6; 595.7)	717.4 (343.9; 1090.8) ***	−29.7 (−354.6; 295.2)
80 year and more	−312.5 (−816.6; 191.5)	201.6 (−231.3; 634.7)	1123.206 (624.1; 1622.2) ***	−250.8 (−729.1; 227.5)
Marital status (ref. other situations)				
Married	653.7 (368.3; 939.1) ***	588.5 (325.8; 851.1) ***	710.0413 (398.0; 1022.0) ***	571.6 (305.0; 838.2) ***
Comorbidity (ref. no comorbidity)				
Medium	355.8 (82.9; 628.7) *	432.4 (182.3; 682.4) **	741.2 (446.4; 1036.0) ***	448.4704 (199.7; 697.1) ***
High	833.4 (453.1; 1213.7) ***	879.7 (538.5; 1220.8) ***	1815.2 (1404.3; 2226.1) ***	886.4 (535.5405; 1237.4) ***
Muscle strength (ref. 1st tertile)				
Second tertile	−988.7 (−1317.5; −659.8) ***			−838.0 (−1150.2; −525.8) ***
Third tertile	−1315.8 (−1669.2; −962.4) ***			−1188.7 (−1522.6; −854.9) ***
BMI	−9.3 (−34.3; 15.7)	−19.2 (−41.064; 2.6)	−14.4 (−40.0; 11.2)	−3.6 (−27.1; 19.7)
Vigorous physical activities (ref. Never)				
Once a week or less	−1244.9 (−1648.4; −841.4) ***	−1222.3 (−1603.0; −841.5) ***		−1042.8 (−1431.5; −654.1) ***
More than ones a week	−1104.8 (−1390.5; −819.1) ***	−1369.2 (−1631.1; −1107.3) ***		−1165.3 (−1435.5; −895.1) ***
Moderate physical activities (ref. Never)				
Once a week or less	−2288.8 (−3020.5; −1557.1) ***	−2593.3 (−3250.1; −1936.6) ***		−2099.6 (−2811.1; −1388.1) ***
More than ones a week	−2505.6 (−3081.2; −1929.9) ***	−2937.186 (−3441.8; −2432.4) ***		−2494.5 (−3049.0; −1940.1) ***
Log income	132.1 (52.8; 211.4) **	−145.5 (−263.3; −27.7) *	−214.1 (−356.3; −72.0) **	−108.8 (−227.2; 9.6)
No smoking	−333.2 (−671.6; 5.2)	−100.4 (−405.8; 205.0)	−363.0 (−734.9; 8.8)	−162.1 (−471.3; 147.0)
No drinking	−248.6 (−548.4; 51.1)	−48.9 (−333.6; 235.7)	−250.3 (−588.0; 87.3)	−10.4 (−293.7; 272.8)
Daily products (ref. <1 times week)				
1–2 times a week	400.2 (−253.0; 1053.6)	625.0 (20.8; 1229.3) *	298.8 (−416.8; 1014.6)	469.9 (−137.1; 1076.9)
3–6 times week/every day	176.8 (−300.3; 654.0)	221.1 (−209.8; 652.2)	27.5 (−506.6; 561.8)	216.9 (−226.6; 660.5)
Legumes, beans or eggs (ref. <1 times week)				
1–2 times a week	85.1 (−211.1; 381.5)	144.3 (−131.5; 420.2)	123.7 (−203.8; 451.3)	129.7 (−147.8; 407.3)
3–6 times week/every day	−114.4 (−390.6; 161.7)	33.5 (−233.3; 300.3)	32.7 (−284.7; 350.3)	4.2 (−264.1; 272.6)
Fruits or vegetables (ref. <1 times week)				
1–2 times a week	165.9 (−732.5; 1064.4)	119.5 (−660.9; 900.1)	138.5 (−779.5; 1056.5)	109.7 (−695.1; 914.7)
3–6 times week/every day	281.0 (−449.2; 1011.3)	157.2 (−470.1; 784.6)	172.2 (−563.2; 907.6)	94.4 (−557.7; 746.6)
Meat, fish or poultry (ref. <1 times week)				
1–2 times a week	−196.9 (−817.0; 423.1)	−7.4 (−549.4; 534.5)	−188.3 (−865.0; 488.3)	−133.6 (−691.4; 424.0)
3–6 times week/every day	−442.2 (−987.3; 102.8)	−19.6 (−493.8; 454.5)	−305.4 (−901.8; 290.8)	−79.7 (−568.9; 409.5)
Country (ref. Austria)				
Germany		−629.4 (−1463.4; 204.6)	−668.2 (−1649.1; 312.6)	−428.6 (−1272.4; 415.2)
Sweden		−2505.4 (−3116.2; −1894.6) ***	−2838.9 (−3548.0; −2129.8) ***	−2165.3 (−2796.0; −1534.6) ***
Netherlands		−1541.1 (−2205.1; −877.195) ***	−1243.4 (−2073.2; −413.6) **	−1283.7 (−1965.2; −602.2) ***
Spain		−2162.8 (−2818.7; −1506.9) ***	−1504.7 (−2314.5; −694.8) ***	−2301.7 (−2970.6; −1632.8) ***
Italy		−1711.0 (−2405.8; −1016.2) ***	−1320.9 (−2147.3; −494.6) **	−1462.6 (−2195.8; −729.3) ***
France		−1908.6 (−2449.9; −1367.3) ***	−1690.4 (−2328.0; −1052.8) ***	−1775.7 (−2328.8; −1222.6) ***
Denmark		−2321.9 (−2929.3; −1714.5) ***	−2606.2 (−3300.8; −1911.6) ***	−2132.7 (−2738.1; −1527.3) ***
Switzerland		−285.3 (−968.8; 398.0)	−393.5 (−1184.0; 396.9)	−271.9 (−955.0; 411.1)
Belgium		−1509.6 (−2065.7; −953.5) ***	−966.0 (−1635.2; −296.8) **	−1530.54 (−2083.2; −977.8) ***
Czech Republic		−2107.3 (−2641.3; −1573.3) ***	−2152.7 (−2782.7; −1522.7) ***	−2051.0 (−2588.8; −1513.2) ***
Slovenia		−2074.7 (−2705.0; −1444.4) ***	−2346.0 (−3076.4; −1615.6) ***	−1810.8 (−2466.0; −1155.5) ***
Estonia		−2970.5 (−3461.5; −2479.6) ***	−3172.0 (−3751.7; −2592.3) ***	−2789.5 (−3285.8; −2293.3) ***

Ref.: reference category; Married refers to ‘Married and living together with spouse’ or ‘Registered partnership’ and the reference category (Other situations) includes: ‘Married, not living with spouse’, ’Divorced’, ‘Widowed’ or ‘Never married’; 95% Confidence Interval in brackets; * *p* < 0.05; ** *p* < 0.01; *** *p* < 0.001.

## Data Availability

This paper uses data from SHARE Waves 4 and 5. Data available on request: https://doi.org/10.6103/SHARE.w4.611, https://doi.org/10.6103/SHARE.w5.611, see Börsch-Supan et al. (2013) for methodological details. The SHARE data collection has been pri-marily funded by the European Commission through FP5 (QLK6-CT-2001-00360), FP6 (SHARE-I3: RII-CT-2006-062193, COMPARE: CIT5-CT-2005-028857, SHARELIFE: CIT4-CT-2006-028812) and FP7 (SHARE-PREP: N°211909, SHARE-LEAP: N°227822, SHARE M4: N°261982).
